# Chimeric virus-like particles presenting tumour-associated MUC1 epitope result in high titers of specific IgG antibodies in the presence of squalene oil-in-water adjuvant: towards safe cancer immunotherapy

**DOI:** 10.1186/s12951-022-01357-1

**Published:** 2022-03-27

**Authors:** Mirosława Panasiuk, Karolina Zimmer, Anna Czarnota, Magdalena Narajczyk, Grażyna Peszyńska-Sularz, Milena Chraniuk, Lilit Hovhannisyan, Sabina Żołędowska, Dawid Nidzworski, Anna J. Żaczek, Beata Gromadzka

**Affiliations:** 1 Intercollegiate Faculty of Biotechnology, University of Gdańsk and Medical University of Gdańsk, A. Abrahama 58, 80-307 Gdańsk, Poland; 2 Laboratory of Electron Microscopy, Faculty of Biology, University of Gdańsk, Wita Stwosza 59, 80-308 Gdańsk, Poland; 3 NanoExpo Sp. z o.o., Kładki 24, 80-822 Gdańsk, Poland; 4 Tri-City Central Animal Laboratory Research and Service Center, Medical University of Gdańsk, Dębinki 1, 80-211 Gdańsk, Poland; 5 Department of in vitro Studies, Institute of Biotechnology and Molecular Medicine, Kampinoska 25, 80-180 Gdańsk, Poland; 6 Institute of Biotechnology and Molecular Medicine, Kampinoska 25, 80-180 Gdańsk, Poland; 7 Laboratory of Translational Oncology, Medical University of Gdańsk, Dębinki 1, 80-210 Gdańsk, Poland; 8 Faculty of Health Sciences, Department of Biochemistry and Molecular Biology, University of Bielsko-Biala, Willowa 2, 43-309 Bielsko-Biala, Poland

**Keywords:** Cancer immunotherapy, Cancer vaccines, VLPs, Bioengineered nanostructures, MUC1

## Abstract

**Background:**

Immunotherapy is emerging as a powerful treatment approach for several types of cancers. Modulating the immune system to specifically target cancer cells while sparing healthy cells, is a very promising approach for safer therapies and increased survival of cancer patients. Tumour-associated antigens are favorable targets for cancer immunotherapy, as they are exclusively expressed by the cancer cells, minimizing the risk of an autoimmune reaction. The ability to initiate the activation of the immune system can be achieved by virus-like particles (VLPs) which are safe and potent delivery tools. VLP‐based vaccines have evolved dramatically over the last few decades and showed great potential in preventing infectious diseases. Immunogenic potency of engineered VLPs as a platform for the development of effective therapeutic cancer vaccines has been studied extensively. This study involves recombinant VLPs presenting multiple copies of tumour-specific mucin 1 (MUC1) epitope as a potentially powerful tool for future immunotherapy.

**Results:**

In this report VLPs based on the structural protein of Norovirus (NoV VP1) were genetically modified to present multiple copies of tumour-specific MUC1 epitope on their surface. Chimeric MUC1 particles were produced in the eukaryotic *Leishmania tarentolae* expression system and used in combination with squalene oil-in-water emulsion MF59 adjuvant to immunize BALB/c mice. Sera from vaccinated mice demonstrated high titers of IgG and IgM antibodies which were specifically recognizing MUC1 antigen.

**Conclusions:**

The obtained results show that immunization with recombinant chimeric NoV VP1- MUC1 VLPs result in high titers of MUC1 specific IgG antibodies and show great therapeutic potential as a platform to present tumour-associated antigens.

**Graphical Abstract:**

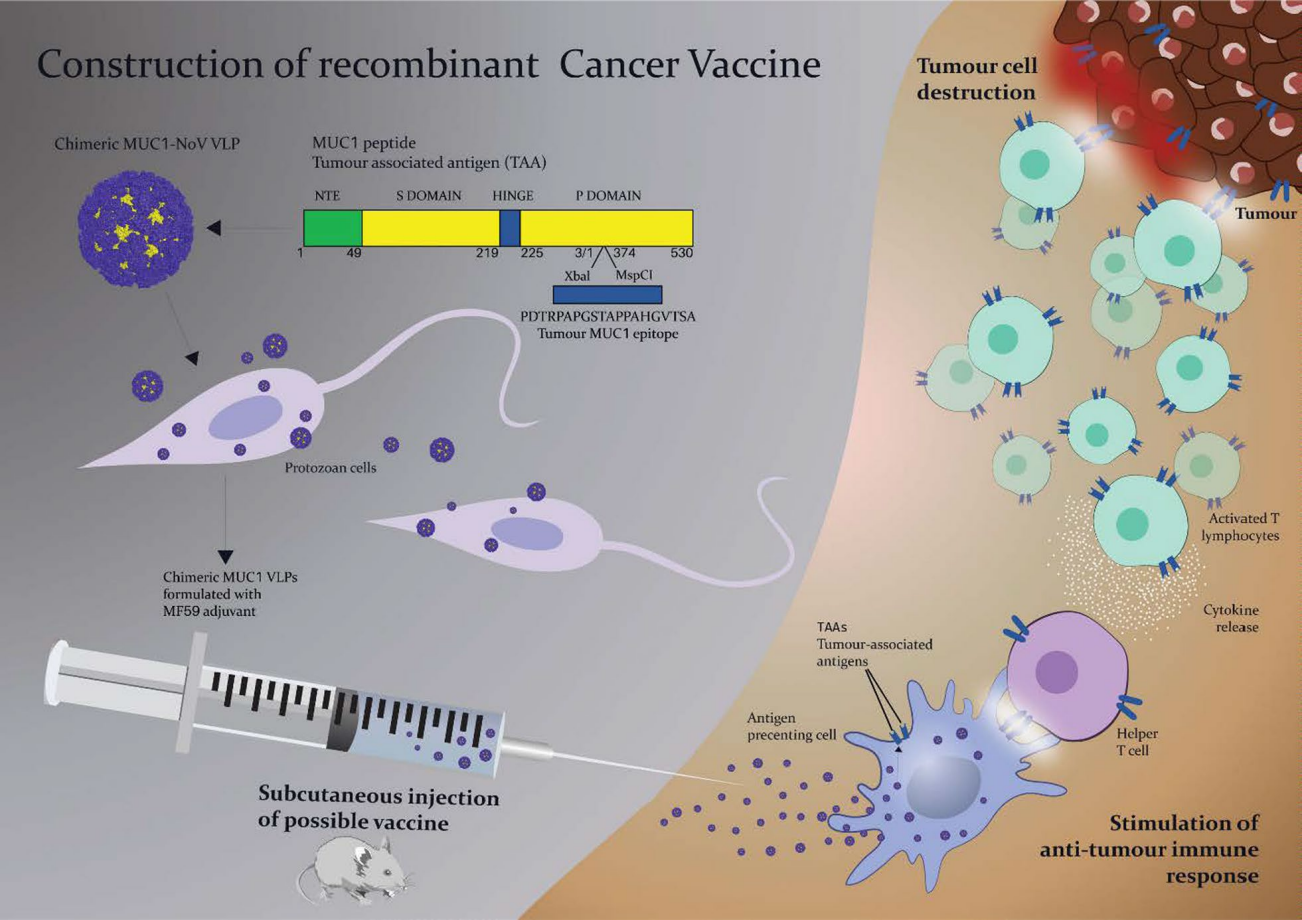

## Introduction

Cancers are a major cause of death worldwide. Chemotherapy, radiotherapy, and surgery, alone or in combination with each other, are the leading methods for cancer treatment so far. In recent years, the use of cancer vaccines is becoming more and more applicable therapy method due to being a highly successful treatment modality. Many diverse vaccine platforms have been evaluated in clinical trials, among which virus-like particles (VLPs) show great potential in cancer vaccine development. VLPs are multimeric nanostructures with a morphology resembling that of native viruses, but devoid of viral genetic material rendering them non-infectious. Research on the protein-based vaccines against cancer has been carried out for many years. However, it is broadly criticized since protein vaccines generally induce relatively stronger levels of humoral than cellular immunity, while the latter is considered essential for tumour immunotherapy. However, because of their small size (20–100 nm), VLPs can travel in the vessels and can be processed to present the specific epitopes of the carried antigen by antigen-presenting cells (APCs) in order to elicit potent specific humoral and cell-mediated immune responses [1]. VLPs are shown to have the ability to initiate the activation of the immune system and are considered strong activators of dendritic cells (DCs) [2]. VLPs represent pathogen-associated molecular patterns (PAMPs) that are recognized by pattern recognition receptors (PRRs) such as toll-like receptors (TLRs) primarily localized on immune cells (e.g., DCs, macrophages) [3, 4]. Additionally, VLPs were shown to overcome the immunosuppressive state of the tumor microenvironment to elicit a strong immune response leading to the destruction of cancer cells [5].

In tumour therapies particular interest is given to the fact that it is possible to induce an immune response in a patient which is directed against his own tumour tissues [6, 7]. From this point of view it is extremely important to be able to identify cancer antigens that are different from the antigens present on healthy cells. Among such antigens, aberrantly glycosylated form of the membrane-bound mucin 1 (MUC1) expressed in multiple types of cancers is considered an attractive target for cancer immunotherapy. Mucins are important glycoproteins which have a protective function for mucosal surfaces [8–11] expressed mainly in most glandular epithelial cells (e.g., breast lung, pancreas, kidney etc.) [12]. It has been shown that cancer cells contain hypoglycosylated form of MUC1 which is distinct from the normal mucin 1 [13]. The structure and function of the MUC1 glycoprotein encoded by *MUC1* gene has been extensively studied confirming the presence of its extracellular, transmembrane and cytoplasmic domains, containing tandemly repeating sequence of 20 amino acids in its extracellular domain [14]. MUC1 is expressed on most epithelial cells and in a healthy tissue negatively charged sugar residues of MUC1 protein create physical barrier to prevent pathogens from colonization [15, 16]. One of the most promising features of MUC1 is its cancer-associated antigenic function due to the presence of tumour-specific antigen epitopes and increased expression in variety of tumour cells [17]. The tumour-associated form of MUC1 is hypoglycosylated [12] in contrast with its heavily glycosylated normal form in healthy, non-cancerous environment which has five potential *O*-glycosylation sites (because of its PDTRP motif) [18, 19]. Due to such altered glycosylation, new epitopes appear on the cell surface that are absent in normal tissues. As a consequence, tumour-associated MUC1 exposes aberrant tumour-associated carbohydrate antigens (TACAs) in combination with the normal antigens [20]. Because of overexpression and hypoglycosylation in cancer environment MUC1 has lost its polarity. Interestingly, the immune system is not able to recognize the tumour-associated hypoglycosylated MUC1 probably because of self-tolerance mechanisms. It has been shown that synthetic MUC1 glycopeptides do not induce an IgG immune response in humans and mouse models if not conjugated with a T-cell stimulating antigen [21]. So far different types of vaccines have been studied, e.g., cell-, DNA-, RNA- and peptide-based vaccines to deliver the MUC1 antigen [22, 23], but the future of next generation cancer vaccines is mostly based on bioengineered nanostructures, such as VLPs. Chimeric VLPs presenting the anti-tumour antigens were described for antigens such as: MUC1, survivin [24], HER-2 [25], carcinoembryonic antigen (CEA) [26], Prostate Specific Antigen (PSA) and more [27–29]. All the developed bioengineered nanoforms were produced in bacterial or insect cells. However, the glycosylation profile of the recombinant proteins and VLPs is very important. Due to the high glycosylation profile of MUC1 and aberrant glycosylation profile of tumour-associated MUC1 it is crucial to choose an expression system supporting the mammalian type of post-translational modification.

Here, for the first time to our knowledge, we present the bioengineered nanostructures based on the Norovirus (NoV) VLPs that present multiple copies of tumour-associated form of MUC1 epitope on their surface. To ensure that the original MUC1 glycosylation profile will be retained, nanostructures were produced in the LEXSY system in *Leishmania tarentolae* (*L. tarentolae*) cells. LEXSY system possesses post-translational machinery equipped with enzymes that carry out the glycosylation pattern of mammalian proteins [30].

Additionally, to further improve the immune response the vaccine preparation was formulated in combination with TLR-independent adjuvant—squalene oil-in-water emulsion MF59 (AddaVax), which is approved to be a safe and effective vaccine adjuvant [31–34].

## Results

### Construction and expression of chimeric VLPs that present MUC1 epitope

**Fig. 1:**
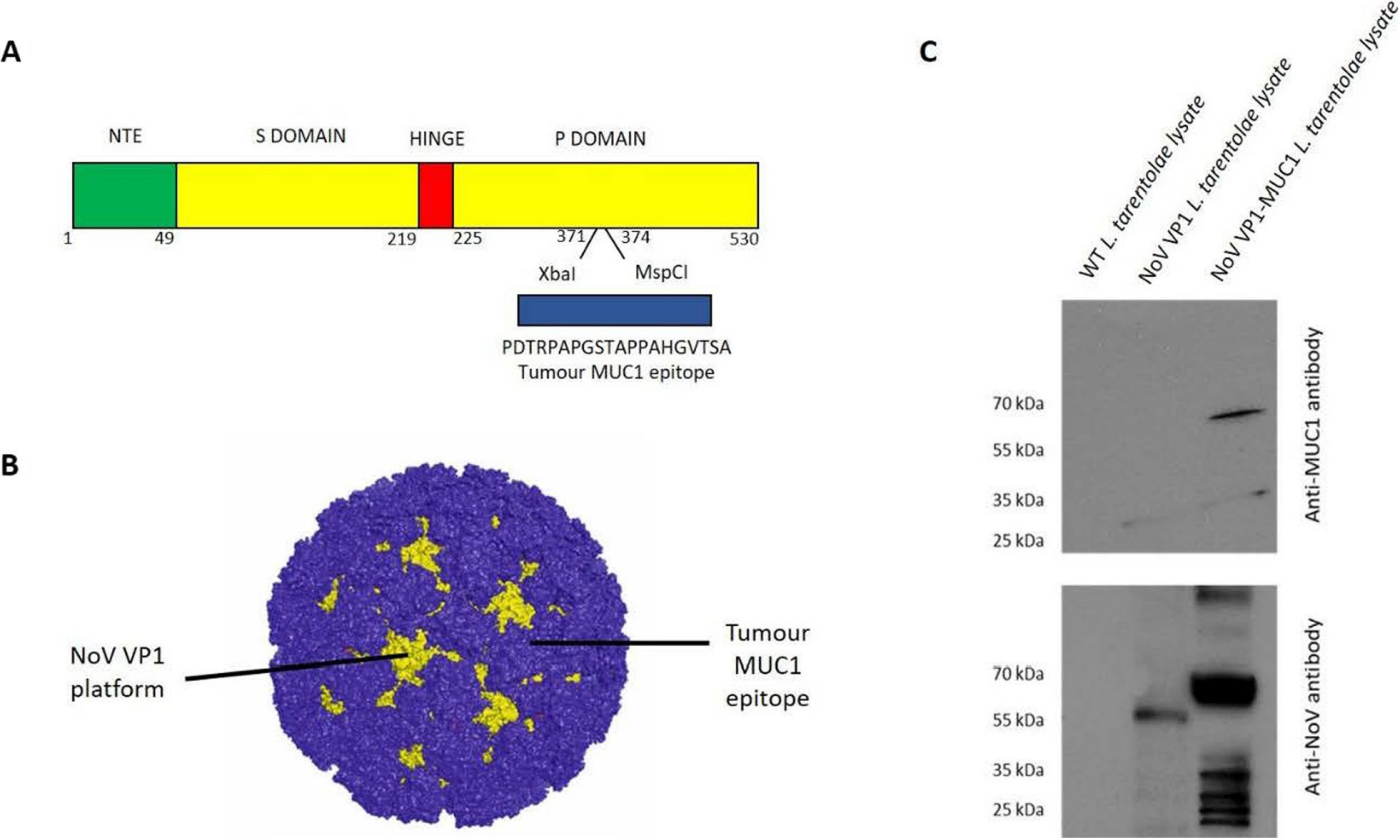
Construction of chimeric VLPs presenting tumour MUC1 epitope. **A** Schematic representation of cloning of tumour-associated MUC1 epitope into NoV *VP1* gene. **B** Predicted presentation of MUC1 epitope on the surface of VLPs. **C** Western blotting analysis of the chimeric NoV VP1-MUC1 expressed in the LEXSY expression system in reducing conditions. The protein was detected as a 70 kDa band in cell lysates using specific anti-MUC1 antibodies and anti-NoV antibodies. WT (wild type) *L. tarentolae* cell lysates and NoV VP1 *L. tarentolae* cell lysates served as controls

NoV major capsid protein VP1 has an ability to self-assemble into VLPs. By methods of genetic bioengineering a sequence of VP1 gene can be modified by inserting foreign epitopes (antigens) [35], thus multiple copies of those epitopes are presented on its surface. In this report we introduce tumour-associated MUC1 epitope (PDTRPAPGSTAPPAHGVTSA) sequence into NoV *VP1* gene to produce MUC1 chimeric VLPs (Fig. 1A, B). The synthetic DNA coding MUC1 epitope was inserted into the second loop of P2 domain of NoV *VP1* gene (Fig. 1A). The expression of the MUC1 chimeric VP1 protein was performed in *L. tarentolae* cells using a tetracycline inducible LEXSY expression system. Protein expression was analysed by western blotting in reducing conditions. As a negative control unmodified NoV VP1 VLPs described before were used [36]. Obtained results confirmed the presence of MUC1 epitope on chimeric VP1-MUC1 protein but not on empty VP1 platform (Fig. 1C). Additionally using anti-NoV antibodies we confirmed the higher molecular weight of chimeric VP1-MUC1 (approximately 70 kDa) in comparison to VP1 platform alone (approximately 60 kDa) (Fig. 1C).

### Production, purification and characterization of chimeric NoV VP1-MUC1 VLPs

**Fig. 2:**
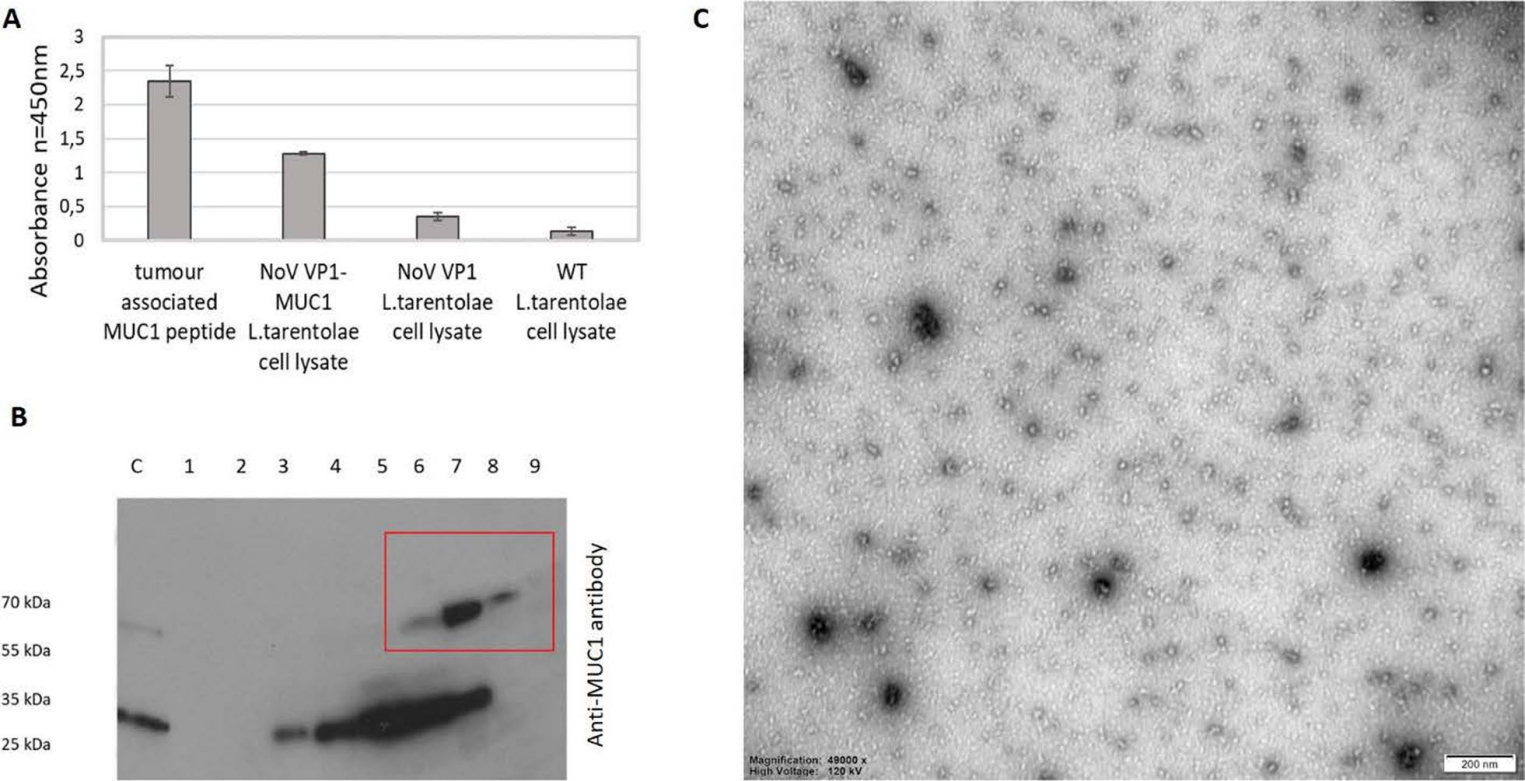
Characterization of chimeric NoV VP1-MUC1 VLPs. **A** Detection of MUC1 epitope in chimeric NoV VP1-MUC1 produced in *L. tarentolae* cell lysates; tumour-associated MUC1 peptide served as a positive control, NoV VP1 *L. tarentolae* cell lysates served as an empty platform control and WT *L. tarentolae* cell lysates as a background control. The bars represent the mean values obtained from triplicate experiments. **B** Purification of chimeric NoV VP1-MUC1 VLPs in OptiPrep gradient. Western blotting analysis of the sequential fractions collected from ultracentrifugation in 30–40% gradient. Protein was detected in cell lysates using specific anti-MUC1 antibodies in fractions 6–8. **C** Electron micrograph of purified *L. tarentolae*-derived chimeric NoV VP1-MUC1 VLPs (scale bar: 200 nm)

The expression of the chimeric NoV VP1-MUC1 VLPs was performed in high-density *L. tarentolae* cell cultures (> 10^8^ cells/ml) using a tetracycline inducible LEXSY expression system. 72 h after induction cells were harvested and lysed. *L. tarentolae* cells expressing chimeric NoV VP1-MUC1 were analysed via ELISA assay using anti-MUC1 antibodies to confirm the ability to present MUC1 epitope. For full analysis chimeric VP1-MUC1 lysates were compared with synthetic MUC1 peptide (positive control), NoV VP1 *L. tarentolae* cell lysates (empty platform) and WT *L. tarentolae* cell lysates (background). MUC1 epitope was specifically detected only in chimeric NoV VP1-MUC1 cell lysates (Fig. 2A).

To determine if the chimeric NoV VP1-MUC1 proteins were in the monomeric, dimeric or multimeric VLP form, we analysed the proteins by OptiPrep (iodixanol) gradient which reported to preserve protein–protein interactions better than concentrated high-osmotic-strength sucrose solutions. Iodixanol gradients have been used with excellent results to purify viral particles whose integrity is sensitive to other separation systems. The lysate containing the chimeric VP1-MUC1 protein from the *L. tarentolae* was analysed in 30–40% gradient followed by fractionation. Each fraction was then analysed by SDS-PAGE and western blotting using anti-MUC1 antibody. Obtained results show clear bands of 70 kDa proteins in fractions 6–8 which correspond to 33% gradient band and is in accordance with passing of particles of approximately 40 nm which is the predicted size of chimeric VP1-MUC1 VLPs. While additional protein bands were detected in other fractions, they did not have the required molecular weight and were most likely partially degraded proteins (Fig. 2B). Purified chimeric VP1-MUC1 VLPs were visualized by transmission electron microscopy (TEM). The obtained NoV VP1-MUC1 VLPs were about 40 nm, which corresponds to the size of the native NoV platform (Fig. 2C).

**Fig. 3:**
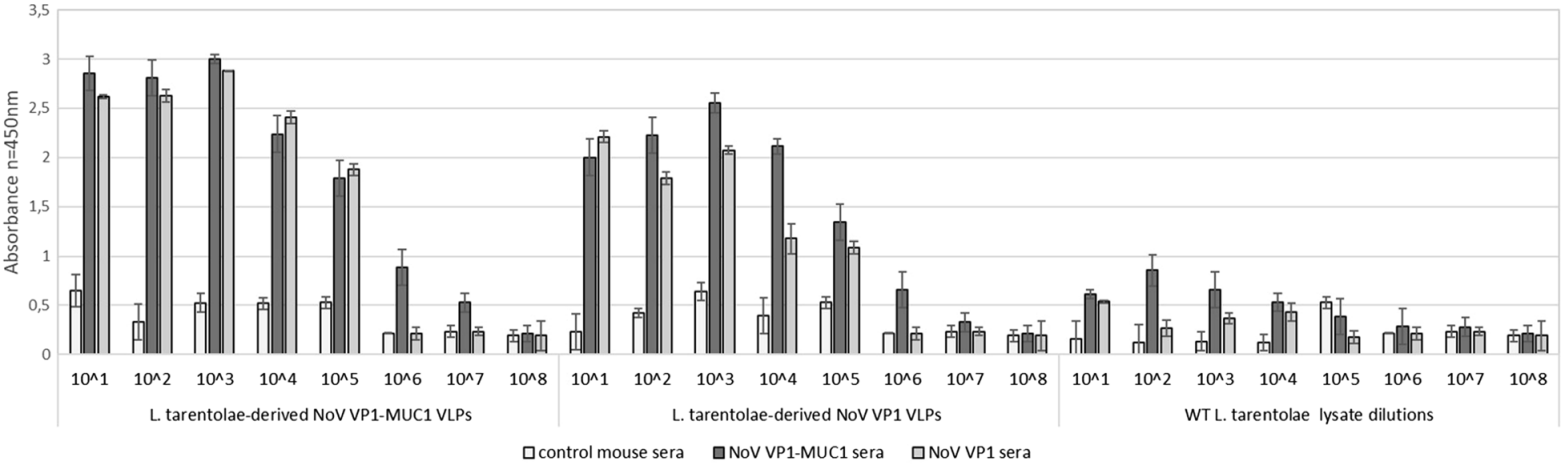
Analysis of the chimeric VLP-MUC1 presenting platform by ELISA assay with anti-MUC1 and anti-NoV antibodies. ELISA plate was coated with serial dilutions of *L. tarentolae* NoV VP1-MUC1, NoV VP1 or WT cell lysates. NoV VP1 *L. tarentolae* cell lysates served as an empty platform control and WT *L. tarentolae* cell lysates as a background control. The dilution factor is depicted on x-axis. For each ELISA assay, the mean from three independent experiments performed is shown. The mean A450 values and standard deviations are shown on the y-axis

To further analyse the chimeric MUC1 VLPs ELISA assay on plates coated with serial dilutions of *L. tarentolae* cell lysates was performed. Immuno assay clearly indicated that the NoV VP1-MUC1 protein expressed in *L. tarentolae* was specifically recognized by anti-MUC1 antibodies (MUC1 was specifically recognized up to 10^3^ lysate dilutions) as well as by anti-NoV antibodies confirming the expression of chimeric platform VP1-MUC1. NoV VP1 *L. tarentolae* cell lysates served as an empty platform control and WT *L. tarentolae* cell lysates as a background control (Fig. 3).

### Mice immunization and analysis of immune response to chimeric NoV VP1-MUC1 VLPs

**Fig. 4:**
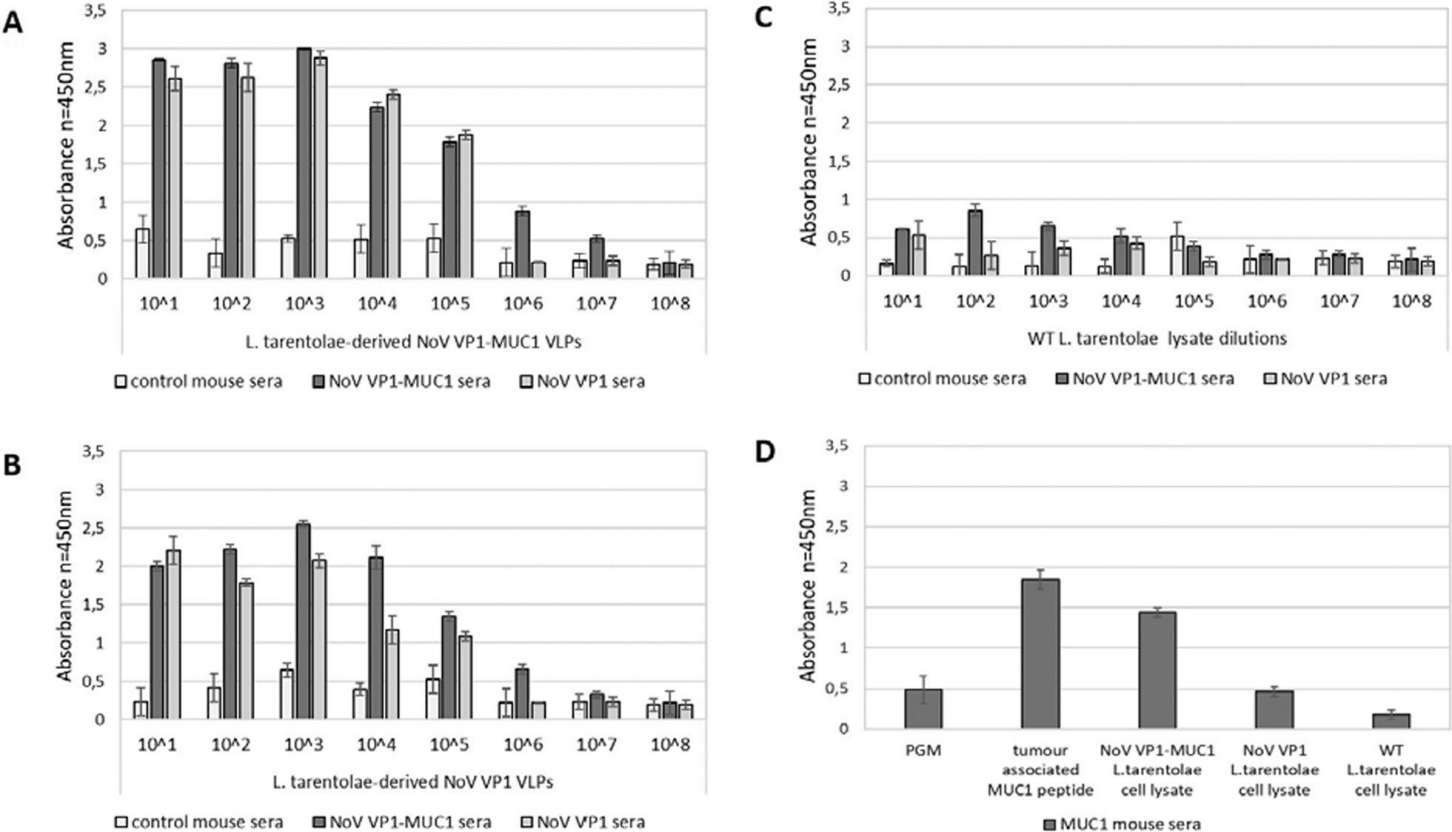
Analysis of the humoral response induced by NoV VP1-based VLPs in BALB/C mice. Recognition of chimeric NoV VP1-MUC1 particles produced in *L. tarentolae* by pooled mouse sera collected after vaccination. ELISA plates were coated with serial dilutions of: **A** recombinant *L. tarentolae* cell lysates containing chimeric NoV VP1-MUC1 VLPs, **B** NoV VP1 VLPs (empty platform) or **C** WT *L. tarentolae* cell lysates (background threshold). For A-C panels the dilution factor of VLPs or WT lysate is depicted on x-axis. **D** Comparison of the detection of MUC1 peptide (positive control) and MUC1 epitope in chimeric NoV VP1-MUC1 expressing *L. tarentolae* cell lysates by MUC1 mouse sera. NoV VP1 *L. tarentolae* cell lysates served as an empty platform control and WT *L. tarentolae* cell lysates as a background control. PGM was used as a control of native mucins. For each ELISA assay, the mean from three independent experiments performed is shown. The mean A450 values and standard deviations are shown on the y-axis

To demonstrate the immunogenicity of chimeric NoV VP1-MUC1 VLPs produced in *L. tarentolae*, three groups of BALB/c mice were immunized subcutaneously on days: 0, 14, and 28 with chimeric NoV VP1-MUC1 VLPs, NoV VP1 VLPs (empty platform) or PBS. All mice were immunized in presence of a squalene-based oil-in-water nanoemulsion adjuvant MF59 (Addavax). Two weeks after the last vaccination, the blood was collected and obtained sera were pooled in each group for further analysis. The humoral response induced by immunization was quantified by ELISA assay on a plate coated with serial dilutions of VLPs (chimeric VP1-MUC, VP1 empty platform and WT lysates). Sera from mice immunized with 1:1 mix of PBS and MF59 adjuvant served as a negative control. The results confirm the ability of VP1-MUC1 sera to recognize both VP1-MUC1 VLPs as well as an empty platform VP1 with high specificity—up to 10^5^ dilution (WT *L. tarentolae* cell lysates served as a background threshold) (Fig. 4A–C). To fully analyse the strength of MUC1 epitope recognition by obtained MUC1 mouse sera the response to native mucins from pig gastric mucous (Porcine gastric mucin, PGM, Sigma Aldrich) and MUC1 epitope in chimeric NoV VP1-MUC1 expressing *L. tarentolae* cell lysates was compared to tumour-associated MUC1 peptide serving as a positive control (Fig. 4D).

**Fig. 5:**
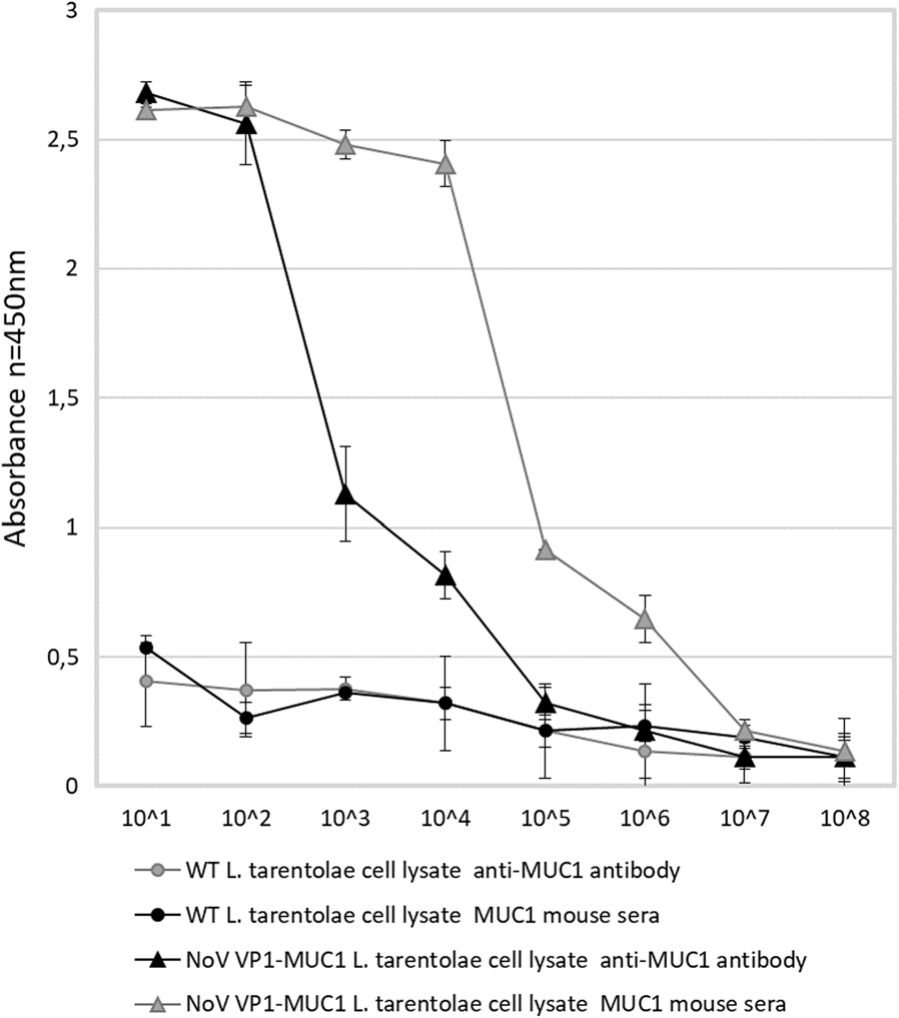
Analysis of the terminal antibody titers in the pooled mouse sera collected after immunization. ELISA plates were coated with *L. tarentolae* cell lysates containing chimeric NoV VP1-MUC1 VLPs or WT cell lysates (background). The dilution factor of the pooled sera is shown on the x-axis. For each ELISA assay, the mean value from three independent experiments performed is presented. The mean A450 values and standard deviations are shown on the y-axis

The end-point serum titrations show that the chimeric NoV VP1-MUC1 antibody titer reached 10^6^ (Fig. 5). The antibody titer was estimated as the serum concentration at which the binding was at least 2 times higher than the background.

### Characterization of antibody isotype response to chimeric VP1-MUC1 VLPs

**Fig. 6:**
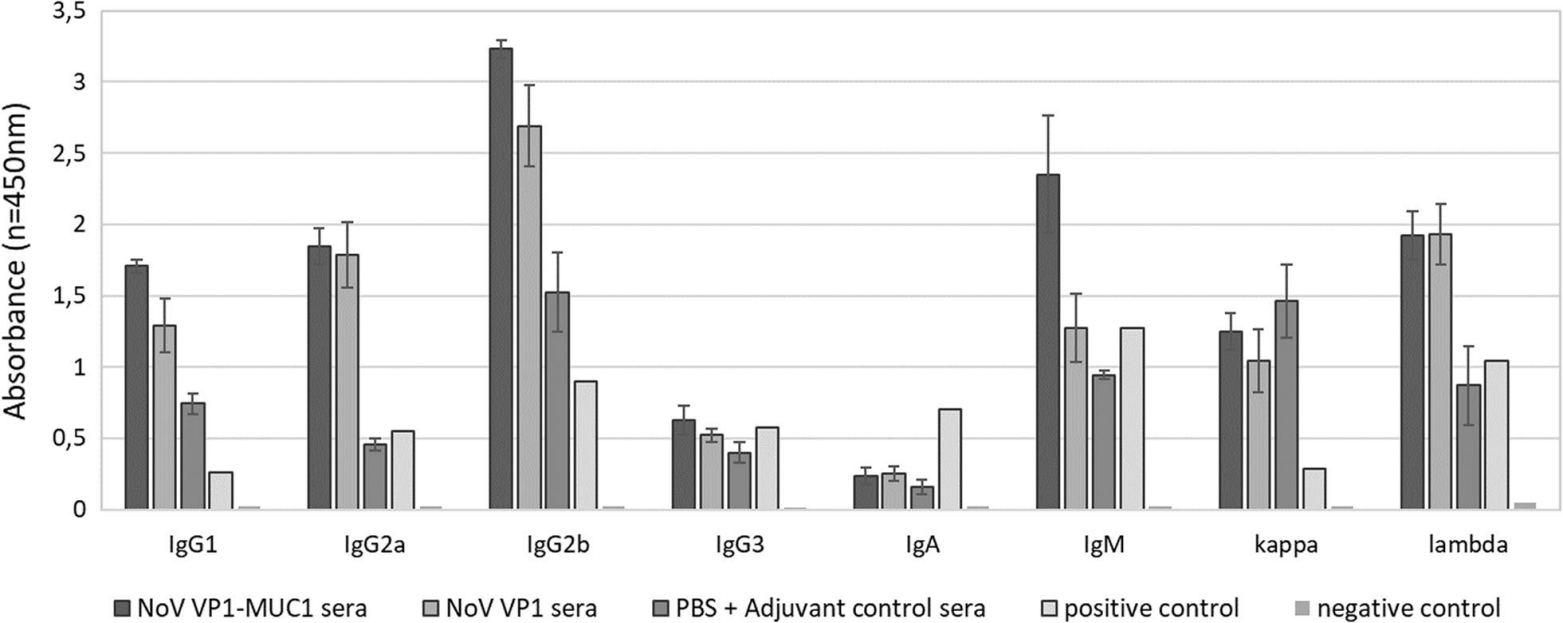
Ig isotyping of the pooled mouse sera collected after immunization. Sera from chimeric NoV VP1-MUC1, NoV VP1 or PBS vaccinated mice were analysed on ELISA plates coated with isotype-specific capture antibodies. Tested Ig isotypes are shown on the x-axis. For each ELISA, the mean value from three independent experiments performed is presented. The mean A450 values and standard deviations are shown on the y-axis

To identify the type of Ig antibodies produced after vaccination, mouse sera were analysed using Ig Isotyping ELISA test (Invitrogen). The analysis showed high levels of IgG1, IgG2a, IgG2b and IgM as well as the presence of both kappa and lambda light chains. Obtained results show strong humoral and cellular response of vaccinated mice to chimeric VLPs presenting tumour-associated MUC1 antigen and the VP1 platform itself. High level of IgG2a suggests the presence of B memory cells. The control group of mice vaccinated with PBS and MF59 adjuvant showed substantial levels of IgG1, IgG2b and IgM confirming immunostimulatory properties of MF59 (Fig. 6). The IgG antibody subclass distribution elicited after vaccination can be used as an indication of the type of immune response, as cytokines produced by Th1 cells promote a cell-mediated immune response, while cytokines produced by Th2 cells trigger the humoral immune response. In mice, the IgG1 subclass is believed to signal a Th2 response, while IgG2a antibodies are associated with Th1-type responses. As shown in Fig. 6 apart from high levels of IgG1 antibodies, the level of obtained IgG2a antibodies was also significant which leads us to believe that vaccination with chimeric MUC1-VLPs was successful in eliciting the humoral response [37, 38].

## Discussion

Despite many advances in the field of medicine, cancer is still one of the leading causes of death worldwide. Among many therapies available, cancer immunotherapies which include cell therapies, antibodies, cytokines, oncolytic viruses, and cancer vaccines are one of the most promising. Standard cancer therapies, such as chemotherapy or radiation, affect patients body system-wide and cause excessive damage to normal tissue. In contrast, immune responses elicited by vaccines specifically target and destroy cancer cells while preserving healthy tissues. Cancer immunotherapies target all stages of neoplastic process (solid tumours, circulating cancer cells, early stages of cancer progression). Importantly, vaccines generate tumour-specific immune memory that provides long-term protection preventing cancer recurrence. Currently, prophylactic cancer vaccines available on the market target oncoviruses such as human papillomavirus and hepatitis B virus. They have successfully prevented cervical cancer and hepatocellular carcinoma [39]. This report is the first to describe the possibility of attaining efficient expression of genetically engineered chimeric NoV-based VLPs presenting TAA in the *L. tarentolae* protozoan. Proposed chimeric VLPs can serve as a possible cancer vaccine candidate and a platform for delivery of tumour associated antigens.

For this study, MUC1 tumour-associated antigen was chosen to be presented on the surface of the chimeric VLPs, as it is considered to be one of the promising targets for cancer vaccines [40]. MUC1 has been shown to be expressed up to tenfold higher on the surface of different human carcinomas than in healthy tissues. Additionally, MUC1-associated antibody production and cellular immune responses have been shown to exert a positive effect on cancer patient outcomes. One of the main features making MUC1 an attractive target for immunotherapies is that in cancer cells, MUC1 is hypoglycosylated, exposing the core epitopes of the extracellular domain that is normally masked by glycans. This exposed VNTR (variable number of tandem repeats) region consists multiple copies of 20 amino acids (PDTRPAPGSTAPPAHGVTSA). Each tandem repeat contains five potential O‑glycosylation sites that act as a potential target for antibody‑mediated therapy. The aberrant glycosylation in this region was shown to be recognized as antigenically distinct by B cells leading to the production of antibodies specifically recognizing tumour-associated altered glycosylation pattern [41]. Therefore, antibodies against tumour-associated MUC1 are binding to the antigen on the surface of cancer cells, and not to normally glycosylated form of MUC1 on the surface of normal cells. Glycans displayed by tumour cells differ from their normal counterparts both in their expression levels and structure. The main alterations of the tumour-associated glycans include increased branching of N-glycans, truncated O-glycan expression, increased levels of sialylation resulting in an increase in sialyl Lewis x (SLe^x^) and Lewisa (SLe^a^) antigens, as well as complex core fucosylation [42, 43]. Due to the complex structures of glycans (even when the protein is aberrantly glycosylated) it still requires proper post-translational cellular machinery to preserve the correct pattern of the displayed glycans. To preserve glycosylation pattern specific for the hypoglycosylated MUC1, chimeric MUC1-VLPs were produced in eukaryotic expression system in protozoan parasite *L. tarentolae*. The major advantage of the LEXSY expression system is its mammalian-type post-translation machinery, which is crucial to preserve specific glycosylation profile of the TAA presented on the nanoparticle. Moreover, LEXSY system allows for easy and cost-efficient scaling up of the cell culture as well as the use of bio-fermenters. These two features make *L. tarentolae*-based expression system attractive for industrial-scale tumour associated antigen production [44]. So far, this expression system was used for production of only a small number of viral antigens, e.g., hemagglutinin [45], human papillomavirus L1 [46], small surface antigen of hepatitis B virus [47] or NoV VLPs [36].

Chimeric NoV VP1-MUC1 protein expressed in *L. tarentolae* cells had higher molecular weight and migrated slower in the reducing SDS-PAGE gel than the NoV VP1 protein (empty platform) which was shown by western blotting analysis. Also, the chimeric VP1-MUC1 protein was specifically recognized by anti-MUC1 antibodies, which was confirmed by ELISA assay. Those results confirm the presence of hypoglycosylated epitope incorporated into NoV VP1 protein. A biophysical analysis of the chimeric NoV MUC1-VLPs performed as an ultracentrifugation in OptiPrep gradient showed that chimeric MUC1 particles were mostly localized in the 33% layer. Moreover, the chimeric VP1-MUC1 protein was properly folded and spontaneously formed VLPs which was confirmed by TEM (Transmission Electron Microscopy) of collected gradient fractions. NoV MUC1-VLPs were spherical in shape and approximately 40 nm in diameter which is consistent with the previously published data about NoV VLPs expression in yeast [48], insect [49] and protozoan cells [36]. A dynamic light scattering analysis (DLS) did not reveal any alterations in the quaternary structure of chimeric MUC1-VLPs in comparison to NoV VLPs – empty platform (data not shown). With advances in recombinant technologies allowing VLPs to serve as a delivery tool or platform for foreign antigens, many VLP‐based cancer vaccines are entering to the stage of clinical development. VLP-based vaccine candidates show to be efficient in stimulation of the immune system against both pathogens and tumour-associated antigens. VLP-based vaccines have the potential to be used as prophylactic or therapeutic cancer vaccines [50] as they can induce both humoral and cellular responses as well as antibody-dependent cellular cytotoxicity (ADCC), which allows effector cells to recognize and kill antibody-coated cells expressing tumour- or pathogen-derived antigens on their surface.

Here, we provide strong evidence of the immunogenic potential of chimeric MUC1-VLPs formed by modified full-length NoV VP1 protein produced in *L. tarentolae*. For mice immunization a formulation of purified chimeric MUC1-VLPs with TLR-independent adjuvant—squalene oil-in-water emulsion MF59 (AddaVax) was used to further boost the immune response to the potential cancer vaccine [51]. Oil-in-water emulsions have been previously shown to increase the efficacy, immunogenicity, and cross-protection of human vaccines as well as enhanced antigen uptake [52–54].

Sera of the mice immunized with chimeric NoV VP1-MUC1 VLPs were able to recognize the protozoan parasite-derived antigens of both chimeric MUC1-VLPs and the empty platform (NoV VP1) while having low reactivity with native mucins. Despite many researchers focus on chimeric VLPs as a delivery platform for foreign antigens, there is no other study showing TAA epitopes presented on the surface of the modified nanoparticles. Additionally, here we present a significantly higher level of obtained anti-MUC1 antibodies which coincides to the results obtained by other research groups [55–57]. Our results demonstrate that immunization with the formulation of chimeric MUC1-VLPs and squalene oil-in-water emulsion MF59 is able to induce a strong immune response and leads to the efficient production of IgG and IgM antibodies. The end-point titration of anti-MUC1 sera showed that antibody titer reached 10^6 and the response to MUC1 antigen was highly specific. The use of VLPs which are naturally occurring conserved PAMPs can stimulate the immune system responses against the delivered antigen. Using this strategy it will be possible to improve the immune response to low immunogenic epitopes such as TAAs [58], thus giving new possibilities for the development of next-generation cancer vaccines.

Presented results show successful expression of the chimeric MUC1 particles in *L. tarentolae* that leads to the production of high titers of tumour-associated MUC1 antibodies. Our findings could be of great importance in the search for an alternative solution serving large-scale nanoparticles and tumour-associated recombinant protein production for pharmaceutical purposes.

## Materials and methods

### Construction of nanostructures presenting multiple copies of MUC1 epitope

DNA coding sequence of MUC1 epitope (PDTRPAPGSTAPPAHGVTSA) was synthesized by Gene Art Gene Synthesis (Thermo Fisher Scientific). The nanostructure, presenting multiple copies of MUC1 epitope, was based on VP1 capsid protein of human NoV. The GII.4 NoV 2012 pandemic variant (Hu/GII.4/Sydney /NSW0514/2012/AU) *Vp1* DNA coding sequence (1637 bp) was modified by addition of cloning sites for XbaI and MspCI restriction enzymes in second loop of P2 domain of VP1 protein, between 371 and 374 amino acids. The sequence was optimized using *L. tarentolae*-adapted codon and synthesized by Gene Art Gene Synthesis (Thermo Fisher Scientific). The synthetized gene was ligated into BglII-NotI restriction sites in the pLEXSY_I-blecherry vector (Jena Bioscience) resulting in platform vector: *pLEXSY_I*-*blecherry3*_VP1. The synthetic epitope was cloned as a XbaI/MspCI fragment into platform vector *pLEXSY_I*-*blecherry3*_VP1. Resulting plasmid *pLEXSY_I*-*blecherry3*_VP1_MUC1 was used for protein expression in LEXSY expression system. As a control *VP1* gen was cloned into BglII-NotI restriction sites in the pLEXSY_I-blecherry vector resulting in vector: *pLEXSY_I*-*blecherry3*_cVP1.

### VP1-MUC1 protein expression in *L. tarentolae*

The inducible LEXSY expression system (Jena Bioscience) was used for NoV VP1-MUC1 protein expression according to the manufacturer’s instructions Briefly, the *pLEXSY_I*-*blecherry3*_VP1_MUC1 plasmid was delivered into *L. tarentolae* cells by electroporation. The electroporated cells were grown in a suspension culture in BHI medium supplemented with bleomycin (100 µg/ml). Subsequently, the recombinant cell line was cultivated in 25 cm^2^ tissue culture flasks filled with the selective medium containing bleomycin at 26 °C and kept in the dark. The T7 promoter-driven transcription was induced by adding tetracycline at the final concentration of 15 µg/ml.

### SDS-PAGE and western blotting

NoV VP1-MUC1, NoV VP1 and WT *L. tarentolae* cell lysates were loaded on 10–20% precast WedgeWell Gel (Thermo Fisher Scientific) and run at the constant voltage of 165 V. After electrophoresis, semi-dry electrotransfer of proteins onto polyvinylidene difluoride membranes was performed. Membranes were then blocked for 1 h in a 5% semi-skimmed milk solution (5%milk/TBS/0,01%Tween20) and incubated overnight with primary antibodies solution: Armenian hamster anti-MUC1 antibody (MA5-11,202, Thermo Fisher Scientific; 1:100 in 5%milk/TBS/0,01%Tween20) or rabbit anti-N terminal capsid protein of NoV antibodies (ab92976, Abcam; 1:1000 in 5%milk/TBS/0,01%Tween20). The following day, the membranes were washed (TBS/0,01%Tween20) and incubated with solution of Peroxidase-conjugated mouse anti-Armenian hamster antibody (Santa Cruz Biotechnology; 1:4000 in 5%milk/TBS/0,01%Tween20) or AffiniPure goat anti-rabbit antibodies (Jackson Immuno Research; 1:4000 in 5%milk/TBS/0,01%Tween20). A reaction was developed with SuperSignal West Pico PLUS Chemiluminescent Substrate (Thermo Fisher Scientific).

### MUC1 presenting VLP production and purification

#### Cell lysis

Tetracycline-induced *L. tarentolae* cell culture was grown in shake flasks for 72 h, at 26 °C, in agitated culture to the final optical density of 4–5 at OD600. Cells were then centrifuged for 15 min, 8000 rpm, at 4 °C. The cell pellet was resuspended in ice-cold lysis buffer (PBS/0,6%Tween-20) and sonicated (for 40 min, 40% amplitude, 10 s time on, 15 s time off). The lysed cells were centrifuged for 40 min, 8000 rpm, at 4 °C, the cell pellet was discarded, and the lysate was left for 16 h at 4 °C to allow particle formation.

#### Ultracentrifugation in a non-ionic iodixanol-based medium gradient (OptiPrep gradient)

The lysate containing VP1-MUC1 VLPs was layered on OptiPrep gradient (Sigma-Aldrich) formed in ultra-clear tube (2 ml of 40% (v/v) OptiPrep, 2,5 ml 36% (v/v) OptiPrep, 2,5 ml 33% (v/v) OptiPrep, 2 ml 30% (v/v) OptiPrep in ultra-clear water) and ultracentrifuged at 27,000 rpm for 16 h at 4 °C. Then, 500 µl fractions were collected and analysed. The OptiPrep buffer was replaced with PBS using Amicon^®^ Ultra 100 K centrifugal units (Merck Millipore). The purity of the fractions was evaluated by SDS-PAGE with InstantBlue (Expedeon) Coomassie based staining.

#### Transmission electron microscopy

Chimeric MUC1 NoV VLPs produced in the protozoan expression system were purified through ultracentrifugation in OptiPrep Gradient and diluted in TM buffer (50 mM Tris HCl, pH 7,4, 10 mM MgCl_2_). In order to visualize, the chimeric VLPs were absorbed on the carbon coated grids followed by negative staining with 2% uranyl acetate. The particles were observed at 120 kV in the Tecnai Spirit BioTWIN (FEI, USA).

#### Detection of MUC1 presenting VLPs using ELISA assay

A 96-well ELISA plate (Pierce Streptavidin High Binding Capacity, Clear) was coated with 100 µl/well of biotinylated PDTRPAPGSTAPPAHGVTSA MUC1 peptide (synthesized by JPT Peptide Technologies) adjusted to 10 µg/ml. The coated plate was incubated for 2 h with shaking at room temperature. Then the plate was washed 4 × 5 min with 200 µl/well of wash buffer (Tris buffered saline pH 7,2/0,1%BSA/0,05%Tween20). Next, 100 µl/well of *L. tarentolae* cell lysates (WT, NoV VP1 and NoV VP1-MUC1 lysates) were used to coat the plate. The plate was incubated for 2 h with shaking in room temperature. The plate was washed as previously. Next, 100 µl/well of Armenian hamster anti-MUC1 antibody (MA5-11,202, Thermo Fisher Scientific; 1:100 in wash buffer) was added and the plate was incubated for 1 h at room temperature, then washed as previously. Then 100 µl/well of Peroxidase-conjugated mouse anti-Armenian hamster antibody (Santa Cruz Biotechnology; 1:2000 in wash buffer) was added and incubated for 1 h at room temperature. The plate was washed as previously and 100 µl/well of HRP-substrate solution was added (1-Step Turbo TMB-ELISA, Thermo Scientific). The plate was incubated in dark until the blue color developed and the reaction was stopped by adding 50 µL of 0,5 M sulfuric acid to each well. Signal intensity was measured at 450 nm using a plate reader (Epoch, Biotek)**.**

#### Characterization of NoV VP1-MUC1 VLPs by ELISA assay

A 96-well ELISA plate (Greiner Microlon High-Binding, clear) was coated with 100 µl/well of sequential dilutions purified *L. tarentolae*-derived VLPs (NoV VP1 and NoV VP1-MUC1). WT *L. tarentolae* cell lysate served as a negative control. The coated plate was incubated overnight at 4 °C. Next, the plate was washed 4 × 5 min with 200 µl/well of washing buffer (PBS/0,05%Tween20) and blocked for 2 h with 250 µl/well of blocking buffer (3%BSA/PBS/0,05%Tween20) at 37 °C. The plate was washed as previously and 100 µl/well of primary rabbit anti-NoV antibodies (Abcam ab92976; in 3%BSA/PBS/0,05%Tween20) and Armenian hamster anti-MUC1 antibody (MA5-11,202, Thermo Fisher Scientific) were added. After incubation the plate was washed as previously, and the appropriate secondary antibody solution (Jackson Immuno Research; in 3%BSA/PBS/0,05%Tween20) was used for detection. Finally, following the last plate-washing step (6 × 5 min with 200 µl/well), 100 µl/well of HRP-substrate solution was added (1-Step Turbo TMB-ELISA, Thermo Scientific), the plate was incubated in dark until the blue color developed, and the reaction was stopped by adding 50 µL of 0,5 M sulfuric acid to each well. Signal intensity at 450 nm was measured using a plate reader (Epoch, Biotek)**.**

### Animal immunization

Two groups of 6 BALB/c male mice, 6 weeks old, were immunized subcutaneously with either 15 µg (day 0) or 10 µg of VP1-MUC1 protein (days: 14, 28, 42) and mixed in the 1:1 ratio with squalene-based oil-in-water nanoemulsion adjuvant MF59 (Addavax, InvivoGen). The mice serving as negative controls were immunized with 1:1 PBS-adjuvant mixture. Additionally, a group of 6 BALB/c male mice **(**6 weeks old) were immunized subcutaneously with (day 0) 10 µg of VP1 protein (days: 14, 28, 42) and mixed in the 1:1 ratio with squalene-based oil-in-water nanoemulsion adjuvant MF59 (Addavax, InvivoGen). All experiments on animals were conducted by an accredited company (Tri-City Central Animal Laboratory Research and Service Center, Medical University of Gdańsk) in accordance with the current guidelines for animal experimentation. The protocols were approved by the Committee on the Ethics of Animal Experiments of the Medical University of Gdańsk (Permit Number: 45/2015). All surgery procedures were performed under isoflurane anaesthesia, and all efforts were taken to minimize animal suffering.

### Analysis of the antibody response

Mouse sera were collected on day 56 and pooled. Antibody response was measured by ELISA assay using (i) 10 µg/ml 100 µl/well of mucins from pig gastric mucous (Porcine gastric mucin, PGM, Sigma Aldrich) in PBS (ii) 10 µg/ml 100 µl/well of biotinylated PDTRPAPGSTAPPAHGVTSA MUC1 peptide (synthesized by JPT Peptide Technologies), (iii) 100 µl/well of *L. tarentolae* cell lysates—WT, (iv) 100 µl/well of *L. tarentolae* cell lysates—NoV VP1 and (v) 100 µl/well of WT *L. tarentolae* cell lysates—NoV VP1-MUC1. The coated plate was incubated for 2 h with shaking at room temperature. Then the plate was washed 4 × 5 min with 200 µl/well of wash buffer (Tris buffered saline pH 7,2/0,1%BSA/0,05%Tween20). Next, 100 µl/well of pooled serum 1:100 was added, and the plate was incubated for 1 h at room temperature, then washed as previously. Then 100 µl/well of Peroxidase-conjugated anti-mouse antibody (Jackson Immuno Research; 1:2000 in 3%BSA/PBS/0,05%Tween20) was added and incubated for 1 h at room temperature. The plate was washed as previously and 100 µl/well of HRP-substrate solution was added (1-Step Turbo TMB-ELISA, Thermo Scientific). The plate was incubated in dark until the blue color developed and the reaction was stopped by adding 50 µL of 0,5 M sulfuric acid to each well. Signal intensity was measured at 450 nm using a plate reader (Epoch, Biotek)**.**

#### End-point titration of NoV VP1-MUC1 mouse sera by ELISA assay

Sera from immunized mice were collected and pooled on day 56 after immunization. A 96-well ELISA plate (Greiner Microlon High-Binding, clear) was coated with 100 µl/well of *L. tarentolae* cell lysates (WT and NoV VP1-MUC1 lysates). The coated plate was incubated overnight at 4 °C. Next, the plate was washed 4 × 5 min with 200 µl/well of washing buffer (PBS/0,05%Tween20) and blocked for 2 h with 250 µl/well of blocking buffer (3%BSA/PBS/0,05%Tween20) at 37 °C. The plate was washed as previously and serial dilutions of pooled mouse sera (in 3%BSA/PBS/0,05%Tween20) were added to the wells and incubated for 1 h at room temperature. Serial dilutions of Armenian hamster anti-MUC1 antibody (MA5-11,202, Thermo Fisher Scientific; in 3%BSA/PBS/0,05%Tween20) served as a positive control. After incubation the plate was washed as previously, and appropriate secondary antibody solution (Jackson Immuno Research; in 3%BSA/PBS/0,05%Tween20) was used for detection. Finally, following the last plate-washing step (6 × 5 min with 200 µl/well), 100 µl/well of HRP-substrate solution was added (1-Step Turbo TMB-ELISA, Thermo Scientific), the plate was incubated in dark until the blue color developed, and the reaction was stopped by adding 50 µL of 0,5 M sulfuric acid to each well. Signal intensity at 450 nm was measured using a plate reader (Epoch, Biotek)**.**

#### Cross-reactivity of NoV VP1-MUC1 mouse sera by ELISA assay

A 96-well ELISA plate (Greiner Microlon High-Binding, clear) was coated with 100 µl/well of sequential dilutions of *L. tarentolae* cell lysates (WT, NoV VP1 and NoV VP1-MUC1 lysates). The coated plate was incubated overnight at 4 °C. Next, the plate was washed 4 × 5 min with 200 µl/well of washing buffer (PBS/0,05%Tween20) and blocked for 2 h with 250 µl/well of blocking buffer (3%BSA/PBS/0,05%Tween20) at 37 °C. The plate was washed as previously and 100 µl/well of NoV VP1-MUC1 serum, NoV VP1 serum or control mouse sera were added and followed by 2 h incubation at room temperature. After incubation the plate was washed as previously, and the appropriate secondary antibody solution (Jackson Immuno Research; in 3%BSA/PBS/0,05%Tween20) was used for detection. Finally, following the last plate-washing step (6 × 5 min with 200 µl/well), 100 µl/well of HRP-substrate solution was added (1-Step Turbo TMB-ELISA, Thermo Scientific), the plate was incubated in dark until the blue color developed, and the reaction was stopped by adding 50 µL of 0,5 M sulfuric acid to each well. Signal intensity at 450 nm was measured using a plate reader (Epoch, Biotek)**.**

#### Characterization of immune response by ELISA isotyping assay

Ig isotyping ELISA test was performed using Ig Isotyping Mouse Uncoated ELISA Kit (Invitrogen) according to manufacturer protocol. Briefly, a 96-well ELISA plate (Greiner Microlon High-Binding, clear) was coated with 100 µl/well of isotype-specific capture antibody in coating buffer according to the suggested plate layout. The plate was sealed and incubated overnight at 4 °C. Next, the plate was washed 4 × 5 min with 200 µl/well of washing buffer and blocked for 2 h with 250 µl/well of blocking buffer at room temperature. The plate was washed as previously and 100 µl/well of mouse sera were added, as well as negative and positive control included in the kit. Mouse sera used for the analysis: NoV VP1-MUC1, NoV VP1 and PBS + MF59 Adjuvant (dilution used—1:1000). The plate was sealed and incubated at room temperature for 1 h on microplate shaker. Next, the plate was washed as previously and 100 µl/well of detection antibody was added. The plate was sealed and incubated at room temperature for 1 h on microplate shaker. Finally, following the last plate-washing step (6 × 5 min with 200 µl/well), 100 µl/well of substrate solution was added and the plate was incubated in dark until the blue colour developed, and the reaction was stopped by adding 100 µL of stop solution. Signal intensity at 450 nm and 570 nm was measured using a plate reader (Epoch, Biotek).

### Statistical analysis

All statistical analyses were performed using Sigmaplot 12.0 software (SYSTAT Software). Statistical differences between the means of the two groups were analysed using t-test. Each experiment was performed in triplicates and the P value p< 0,05 was considered to be statistically significant.

### NoV VP1 internal controls

Purified NoV VP1 VLPs produced in *L. tarentolae* cells (empty epitope presenting platform) and NoV VP1 sera obtained after mouse immunization described previously [36] served as internal controls for all the experiments.

## Data Availability

The datasets used and/or analysed during the current study are available from the corresponding author on reasonable request.
